# Multi-susceptibile Single-Phased Ceramics with Both Considerable Magnetic and Dielectric Properties by Selectively Doping

**DOI:** 10.1038/srep09498

**Published:** 2015-04-02

**Authors:** Chuyang Liu, Yujing Zhang, Jingguo Jia, Qiang Sui, Ning Ma, Piyi Du

**Affiliations:** 1 State Key Laboratory of Silicon Materials, Department of Materials Science and Engineering, Zhejiang University, Hangzhou 310027, P.R. China

## Abstract

Multiferroic ceramics with extraordinary susceptibilities coexisting are vitally important for the multi-functionality and integration of electronic devices. However, multiferroic composites, as the most potential candidates, will introduce inevitable interface deficiencies and thus dielectric loss from dissimilar phases. In this study, single-phased ferrite ceramics with considerable magnetic and dielectric performances appearing simultaneously were fabricated by doping target ions in higher valence than that of Fe^3+^, such as Ti^4+^, Nb^5+^ and Zr^4+^, into BaFe_12_O_19_. In terms of charge balance, Fe^3+^/Fe^2+^ pair dipoles are produced through the substitution of Fe^3+^ by high-valenced ions. The electron hopping between Fe^3+^ and Fe^2+^ ions results in colossal permittivity. Whilst the single-phased ceramics doped by target ions exhibit low dielectric loss naturally due to the diminishment of interfacial polarization and still maintain typical magnetic properties. This study provides a convenient method to attain practicable materials with both outstanding magnetic and dielectric properties, which may be of interest to integration and multi-functionality of electronic devices.

Nowadays, the integration and multi-functionality are important goals in the area of developing high quality electronic devices. To contribute multi susceptibilities such as magnetic and dielectric properties simultaneously, percolative ceramic composites composed of ferroelectric and ferromagnetic phases have been extensively studied in recent decades^1^,^2^,^3^,^4^,^5^,^6^. The percolative composites are impressive to contribute both extraordinary dielectric and magnetic performances, because the paradox caused by composite law is solved^7^,^8^. However, the mismatched interfaces or grain boundaries originating from the inevitable contact between dissimilar lattice structures will be introduced, thus deficiencies as well as space charges will appear in the composite ceramics. The dielectric loss determined by Maxwell Wagner effect will increase considerably sometimes to ~1^9^, which implies that it is of great importance to eliminate the mismatch and diminish imperfect interfaces to decrease the dielectric loss.

Single-phased ceramic materials are naturally thought to be candidates with relatively matched interfaces and low dielectric loss. Herein, single-phased BaFe_12-x_Ti(Nb, Zr)_x_O_19_ ceramics were fabricated by a sol-gel process. It is worth noting that BaFe_12_O_19_ is a typical ferrite and has excellent magnetic properties. Ti^4+^, Nb^5+^ and Zr^4+^ are universal doping ions with higher electron valence than that of Fe^3+^. In this case, Ti^4+^, Nb^5+^ and Zr^4+^ ions are found to replace Fe^3+^ ions due to their close ionic radii and valance state, making part of neighbor Fe^3+^ ions transfer into Fe^2+^ ions for charge balance^10^. The electron hopping between Fe^3+^ and Fe^2+^ ions form electron pair dipoles(Fe^3+^/Fe^2+^ pairs), which will cause colossal permittivity^11^. Dielectric loss is decreased, which is exactly lower than that of the percolative composites, due to the elimination of the mismatched grain boundaries and thus diminishment of space charges in this case. In this work, the simultaneous advent of colossal permittivity, excellent magnetic properties and low losses suggests that the single-phased ferrite ceramics doped by target ions are potential multifunctional ceramic candidates.

## Results and Discussion

### Formation of Fe^2+^ ions and Fe^2+^/Fe^3+^ pairs in the ferrites with doping Ti^4+^

**Figure 1:**
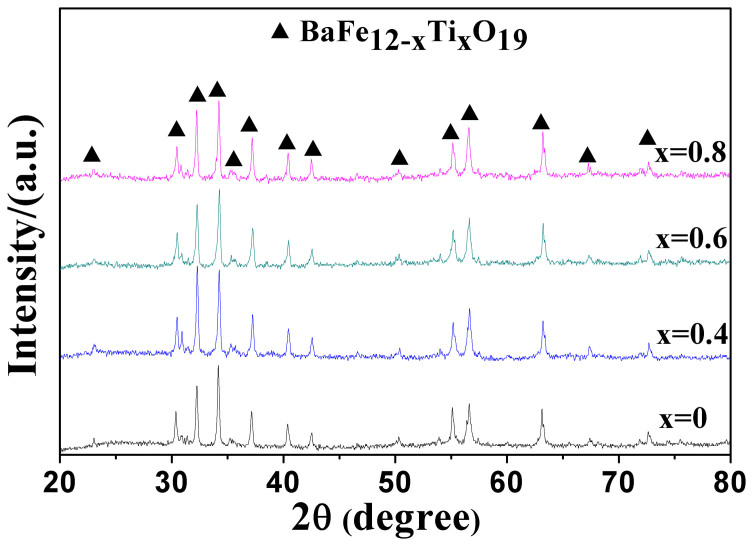
XRD patterns of barium ferrite ceramics BaFe_12-x_Ti_x_O_19_ (x = 0, 0.4, 0.6 and 0.8) sintered at 1200°C for 3 h.

Fig. 1 illustrates the XRD patterns of the barium ferrite ceramics. As can be seen, only M-type barium ferrite phase is detected in all the samples. The structural parameters ‘a’ and ‘c’ as well as cell volume of barium ferrite ceramics are listed in Table 1. It is found that lattice constants and cell volume both decrease initially from x = 0 to x = 0.4 and then increase slowly with Ti^4+^ ions increasing. The smallest “a” and “c” are obtained when x = 0.4.

**Figure 2:**
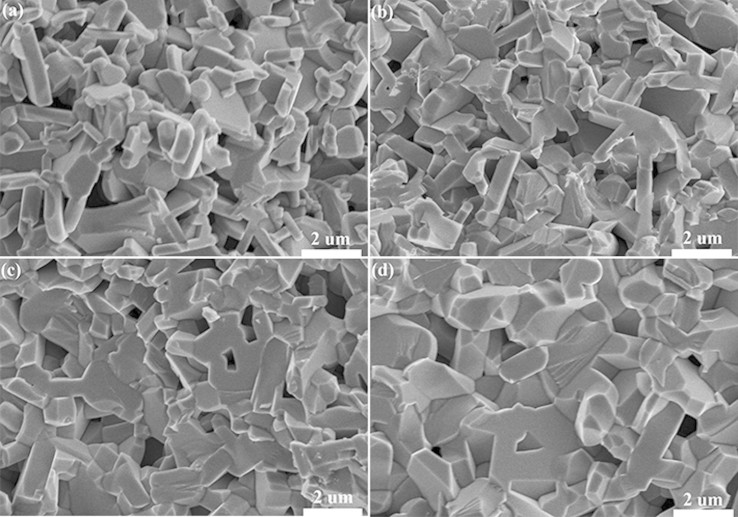
SEM photographs of barium ferrite ceramics of (a) BaFe_12_O_19_, (b) BaFe_11.6_Ti_0.4_O_19_, (c) BaFe_11.4_Ti_0.6_O_19_ and (d) BaFe_11.2_Ti_0.8_O_19_ sintered at 1200°C for 3 h.

Fig. 2 shows SEM photographs of the barium ferrite ceramics. It is seen that the ceramics with or without Ti^4+^ doping all form typical hexagonal plate-like particles. The grain size seems to become larger when Ti^4+^ content increases from x = 0 to x = 0.8 as shown from Fig. 2(a) to Fig. 2(d), which indicates that the formation and grain growth of barium ferrite ceramics are promoted apparently with increasing Ti^4+^ content.

The lattice constants of the ferrites decrease initially and then increase slowly with addition of Ti^4+^. As is known, Ti^4+^ ions added into the ceramic matrix probably substitute for constituent Fe^3+^ ions in barium ferrite due to the close ionic radius of Ti^4+^ (0.605 Å) and Fe^3+^ (0.645 Å)^12^, which has been evidenced by Mössbauer spectroscopy elsewhere^13^. Meanwhile, defect reaction as following will be triggered.
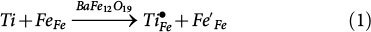
It is seen that some Fe^3+^ ions will be transferred to be Fe^2+^ ions in terms of charge balance. In fact, there are many deficiencies such as oxygen vacancies with positive charge exist in barium ferrite ceramics originally^14^. When Ti^4+^ ions are initially doped into barium ferrites, for electronic balance, the oxygen vacancies may be restrained to form dominantly rather than the transformation of Fe^3+^ into Fe^2+^ in the ferrites. Therefore, the lattice constants are reasonable to decrease when Ti^4+^ ions are doped into the ferrites initially due to the smaller size of Ti^4+^ compared with Fe^3+^. Then, as oxygen vacancies reach smallest, Fe^2+^ ions will start to be produced largely based on defect reaction (1) with Ti^4+^ ions continuously doping into barium ferrites. Because Ti^4+^ has a little smaller radius and Fe^2+^ (0.78 Å) has a much larger radius than Fe^3+^^12^, the lattice constants of the barium ferrites increase probably with increasing Ti^4+^ content. In this case, lattice constants as well as cell volume start to increase when Ti^4+^ content reaches x = 0.6. It indicates that a large number of Fe^2+^ ions are generated in BaFe_12-x_Ti_x_O_19_ with x ≥ 0.6.

**Figure 3:**
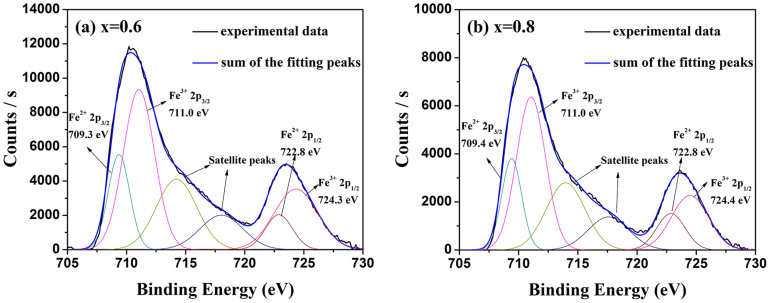
The XPS spectra of Fe 2p for (a) BaFe_11.4_Ti_0.6_O_19_ and (b) BaFe_11.2_Ti_0.8_O_19_ sintered at 1200°C for 3 h.

**Figure 4:**
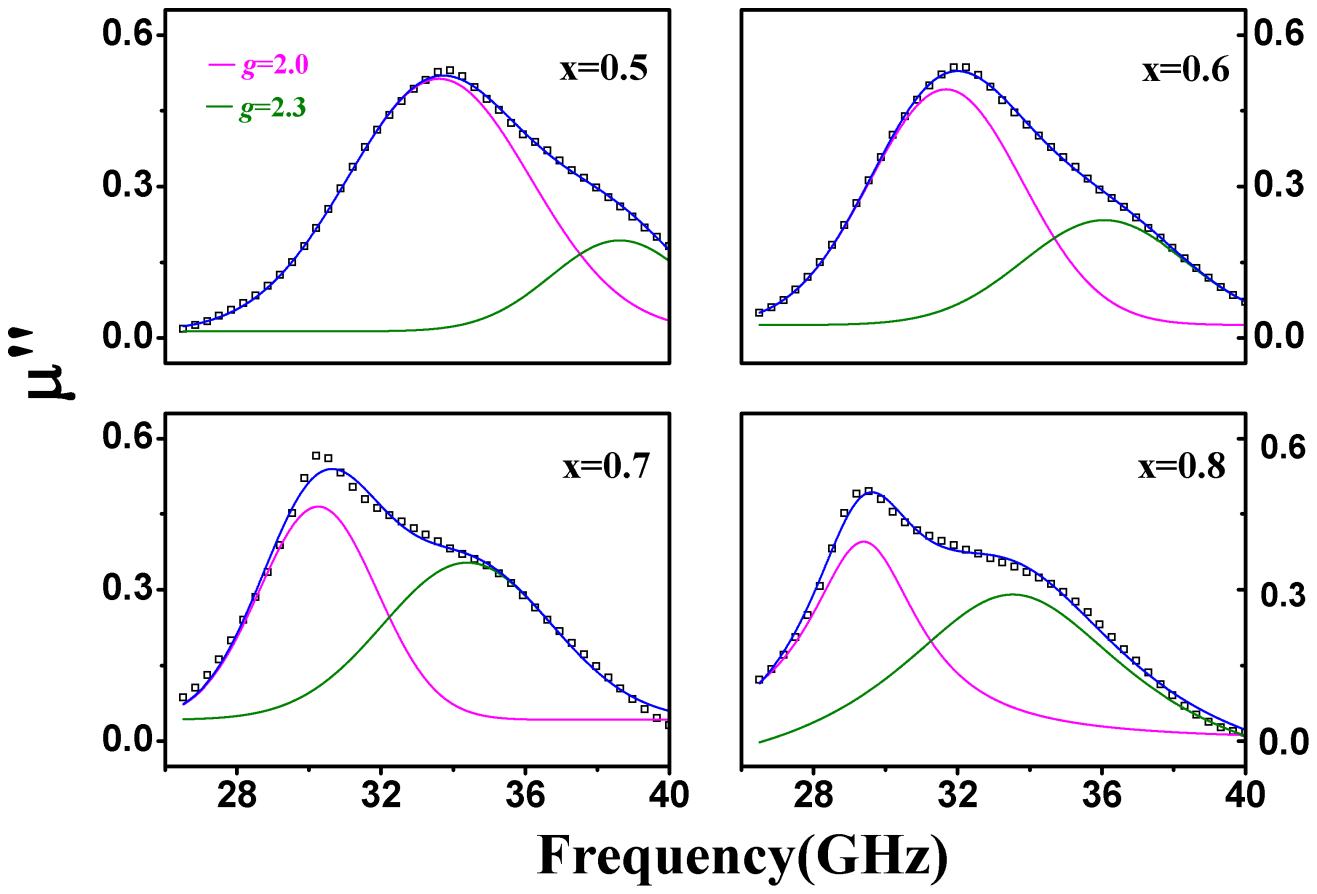
Imaginary part (*μ*″) of the relative complex permeability of BaFe_12-x_Ti_x_O_19_ (x = 0,5 0.6, 0.7 and 0.8) sintered at 1200°C for 3 h.

Moreover, Fig. 3 demonstrates the XPS spectra of Fe 2p for BaFe_11.4_Ti_0.6_O_19_ (Fig. 3a) and BaFe_11.2_Ti_0.8_O_19_ (Fig. 3b) after subtracting baseline, in which C1s peak at 285 eV was used for charge correction and the peaks at ~709.3 eV and ~722.8 eV are belong to Fe^2+^ 2p_3/2_ and Fe^2+^ 2p_1/2_, respectively^15^. It is apparent that Fe^2+^ ions are formed in the ferrites with Ti doping of x ≥ 0.6 which is in fact related with the transformation between Fe^3+^ and Fe^2+^ ions based on Eq. (1). For clearly understanding, it can be confirmed as shown in Fig. 4, in which there are two resonance peaks with two Landé factors (*g*) of 2.0 and 2.3 appearing in imaginary part (*μ*″, the magnetic loss) of the relative complex permeability of BaFe_12-x_Ti_x_O_19_ (x = 0.5, 0.6, 0.7 and 0.8) over 26.5–40 GHz^10^. As a matter of fact, the *g* of around 2.3 is from the exchange coupling between Fe^3+^ and Fe^2+^ ions (Fe^3+^/Fe^2+^ pairs) which is different from ~2.0 of Fe^3+^ in the ferrites^10^. The intensity of the peak about Fe^3+^/Fe^2+^ pairs increases apparently with increasing Ti^4+^ ions. So in this work, Fe^3+^/Fe^2+^ pairs with Fe^2+^ ions generated by doping Ti^4+^ are clearly formed in BaFe_12-x_Ti_x_O_19_ especially with x ≥ 0.6.

### Magnetic properties kept high effectively in the doping ferrite

**Figure 5:**
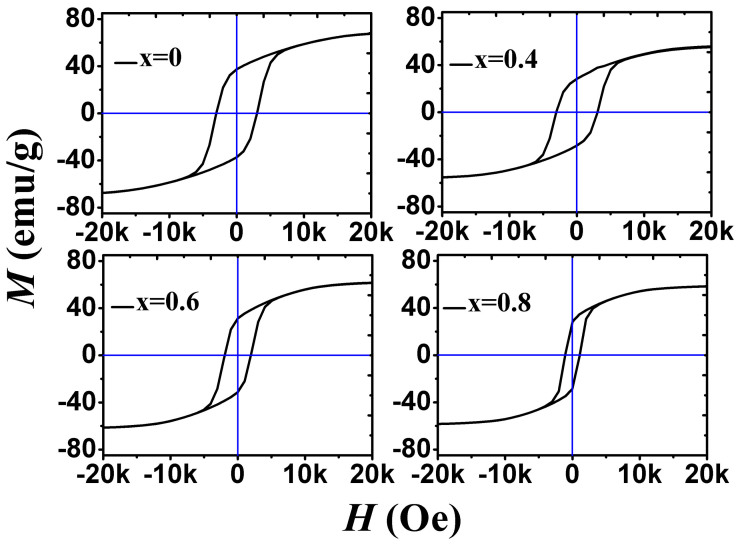
Hysteresis loops of the BaFe_12-x_Ti_x_O_19_ (x = 0, 0.4, 0.6 and 0.8) sintered at 1200°C for 3 h.

Fig. 5 displays hysteresis loops of the BaFe_12-x_Ti_x_O_19_ ferrites (x = 0, 0.4, 0.6 and 0.8). The data of coercive force (*H*_c_), anisotropic field (*H*_a_), saturation magnetization (*M*_s_) and residual magnetization (*M_r_*) deduced from Fig. 5 are summarized in Table 2. It is seen that the maximum *H_c_*, *H*_a_, *M*_s_, *M*_r_ and area of hysteresis loop appear in BaFe_12_O_19_ and they tend to decrease gradually with increasing Ti^4+^ content. *M_s_* and *H_a_* of all the ferrits are obtained through the law of approach to saturation (LAS), which can be expressed as Eq. (2)^16^:
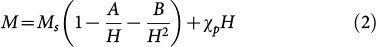
where *A* is the inhomogeneity parameter, *B* is the anisotropy parameter and *χ*_p_ is the high-field differential susceptibility. *B* of hexagonal symmetry can be expressed as Eq. (3).
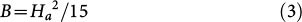


It is shown in Table 2 that *H_a_* of BaFe_12-x_Ti_x_O_19_ decreases dramatically from 15.43 kOe to 10.43 kOe as x varies from 0 to 0.8. In fact, grain size increases and thus the amount of grain boundaries will decrease with increasing Ti^4+^ ions in the ferrites as shown in Fig. 2. Fe^3+^ ions which in general contributes *H_a_* in barium ferrite is substituted by non-magnetic Ti^4+^ ions in this case and hence *H_a_* will decrease. The more the Fe^3+^ ions are replaced, the weaker the *H_a_* is Ref. 16. The *H_a_* of BaFe_12-x_Ti_x_O_19_ decreases therefore with increasing Ti^4+^ content to x = 0.8 although the reduction in grain boundaries will increase *H_a_*. In addition, coercivity which represents the ability to resist magnetization reversal process under a reverse magnetic field is mainly controlled by the hindrance for nucleation of reverse domain, domain wall motion and spin rotation in magnetic materials^17^. As *H_a_* which impedes spin rotation decreases with increasing Ti^4+^ ions in the ferrites, the coercivity decreases hence with increasing Ti^4+^ ions^18^. Moreover, nuclei of reverse domains are easily formed around the deficiencies such as grain boundaries and dislocations, while the deficiencies can also act as pinning centers to hinder domain wall motion. In fact, grain boundaries have a marked influence on pinning domain wall instead of inducing nucleation of reverse domains in barium ferrite^19^. The coercivity of barium ferrite will decrease significantly with decreasing grain boundaries (increasing grain size). Due to the decrease in both *H_a_* and grain boundaries, the coercivity decreases eventually from 3.04 kOe to 1.06 kOe with increasing Ti^4+^ content from x = 0 to x = 0.8, which is about 65% lower than that of undoped one. It implies that the Ti^4+^ doped barium ferrite ceramics will be a good candidate with low energy consumption applied in devices.

Meanwhile, *M_s_* and *M_r_* still maintain high values in BaFe_11.2_Ti_0.8_O_19_. In fact, Fe^3+^ ions of BaFe_12_O_19_ in 12k, 2a, and 2b are up-spin and 4f_1_ and 4f_2_ are down-spin^20^. In this case, the net magnetization of the ferrites is contributed by excess of up-spin magnetic moments. That is to say, if Fe^3+^ ions are substituted by non-magnetic Ti^4+^ ions in 12k, 2a, and 2b sites, magnetization will decrease. Conversely, magnetization will be improved if Fe^3+^ ions which are in 4f_1_ and 4f_2_ sites are substituted. As is reported, Ti^4+^ substitutes preferably for Fe^3+^ in 12k, 2b, and 4f_2_ sites with both states of up spin and down spin^21^. It leads to only a gentle decline of both saturation and residual magnetization in the ferrites with increasing Ti^4+^. Apparently, *M_s_* and *M_r_* of BaFe_11.2_Ti_0.8_O_19_ of about 60 emu/g and 29 emu/g, which are only 20 ~ 30% lower than that of undoped one, are still high enough in practical use in devices keeping typical magnetic properties.

**Figure 6:**
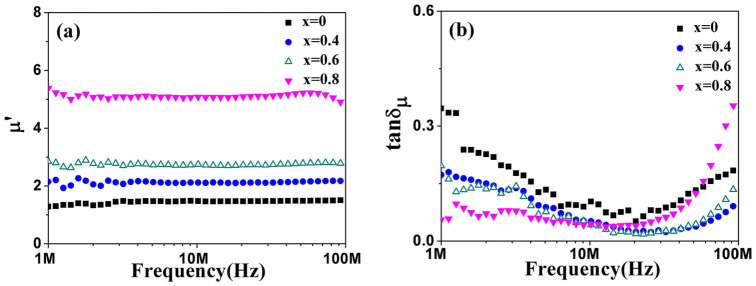
(a) Permeability and (b) Magnetic loss tangent of BaFe_12-x_Ti_x_O_19_ ceramics (x = 0, 0.4, 0.6 and 0.8) sintered at 1200°C for 3 h.

Fig. 6(a) and 6(b) shows the permeability and magnetic loss tangent of the BaFe_12-x_Ti_x_O_19_ (x = 0, 0.4, 0.6 and 0.8) ceramics as a function of frequency respectively. It is seen that permeability of all the samples is almost independent of frequency, except for a little bit decrease in BaFe_11.2_Ti_0.8_O_19_ above 70 MHz. Meanwhile, the frequency independent permeability increases rapidly from about 1.5 to 5.1 with x varying from 0 to 0.8. The magnetic loss tangent of the ferrites depends on frequency and Ti^4+^ content. It decreases from ~0.35 to ~0.07 at low frequency of 1 MHz and increases from ~0.1 to ~0.4 around 100 MHz respectively with increasing content of Ti^4+^ ions from x = 0 to x = 0.8. While it is as low as ~0.1 at moderate frequency.

Obviously, the permeability improves with increasing Ti^4+^ ions in barium ferrites. As is known, domain wall motion and domain rotation are two dominant magnetization processes for polycrystalline ferrites^22^. For the BaFe_12-x_Ti_x_O_19_ polycrystalline ceramics, grain boundaries abating due to larger grain size in high Ti^4+^ doped samples promotes domain wall motion^23^. Furthermore, anisotropic field and demagnetizing field impeding domain rotation in the ferrites decreases with increasing Ti^4+^ content^24^. Consequently, controlled by enhancing both domain wall motion and spin rotation, the permeability of BaFe_11.2_Ti_0.8_O_19_ ceramic is improved to 5.1, which is 3 ~ 4 times of BaFe_12_O_19_ ceramic.

Except for the permeability, magnetic loss is also related naturally to the doping content in the ferrites. Considering the high electrical resistivity of barium ferrite, eddy loss can be neglected^10^. Magnetic loss of BaFe_12-x_Ti_x_O_19_ over the frequency range between 1 MHz and 100 MHz is contributed dominantly by hysteresis loss and residual loss^25^. Actually, hysteresis loss is predominant over lower frequency range, while residual loss takes charge in higher frequency range^26^. As can be seen in Fig. 5, area of hysteresis loops reduces with increasing Ti^4+^ ions content. The magnetic loss decreasing with Ti^4+^ content at low frequency is thus controlled by hysteresis loss, which is ~0.35 of BaFe_12_O_19_ to ~0.07 of BaFe_11.2_Ti_0.8_O_19_ at ~1 MHz. However, the residual loss is contributed in general by domain wall resonance which occurs at frequency above 100 MHz in BaFe_12_O_19_^27^. As shown in Fig. 6(b), it seems that the resonance peak moves toward lower frequency range with doping Ti^4+^ in the ferrites. It implies that the magnetic loss of the BaFe_12-x_Ti_x_O_19_ ceramics is importantly controlled by the residual loss, which increases a little from ~0.1 to ~0.4 with doping Ti ions from x = 0 to 0.8 at ~100 MHz. At moderate frequency, magnetic loss is as low as ~0.1, which is a little bit decrease with doping Ti in the ferrites due to a little reduction of hysteresis loss. Obviously, the magnetic loss of BaFe_12-x_Ti_x_O_19_ ceramics is diminished to be lower than ~0.1 with increasing Ti^4+^ content to x = 0.8, especially at frequencies below 70 MHz.

### Colossal permittivity and low dielectric loss of the doped single-phased ferrites

**Figure 7:**
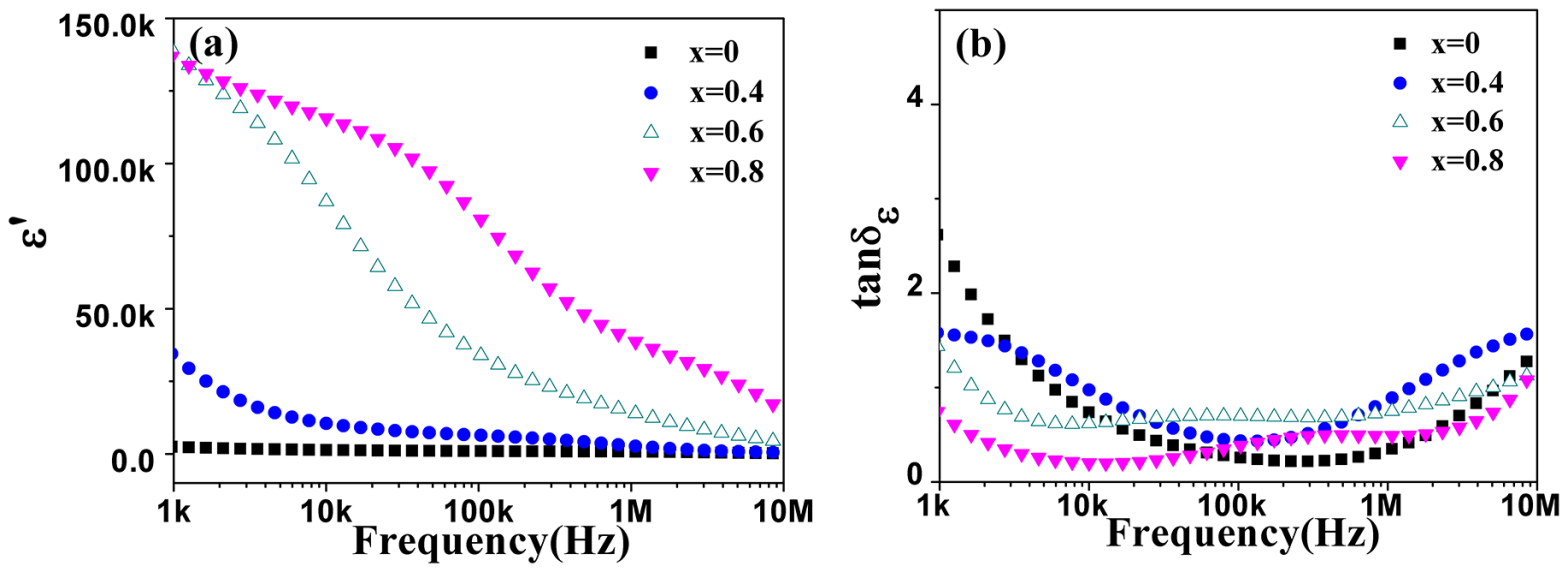
(a) Permittivity and (b) Dielectric loss tangent of BaFe_12-x_Ti_x_O_19_ ceramics (x = 0, 0.4, 0.6 and 0.8) sintered at 1200°C for 3 h.

Fig. 7(a) and 7(b) shows the permittivity and dielectric loss tangent of the BaFe_12-x_Ti_x_O_19_ (x = 0, 0.4, 0.6 and 0.8) ceramics as a function of frequency respectively. The permittivity of the ceramics increases significantly at low frequency range with doping Ti^4+^. Unlike the permittivity of barium ferrite without doping which is almost independent of frequency, the permittivity of the Ti^4+^ ions doped barium ferrites decreases rapidly with increasing frequency and the decreasing speed becomes slow as the content of Ti^4+^ is high from x = 0.4 ~ 0.6 to x = 0.8. A steplike shoulder appears typically in permittivity at moderate frequency with Ti content of x ≥ 0.6. Colossal permittivity which is about 100 k below 100 KHz and 20 k above 1 MHz appears in BaFe_11.2_Ti_0.8_O_19_ ceramic. The dielectric loss tangent of BaFe_12-x_Ti_x_O_19_ ceramics decreases dramatically initially and then keeps stable with increasing frequency and it reduces accordingly with rising Ti^4+^ ions in the ferrites.

It is known that space charge polarization contributes most probably high permittivity especially at low applied frequencies^28^. Free electric charges may easily increase with doping and move freely in ceramics without localization. It will contribute the permittivity to the ceramics due to the charge response and the permittivity thus decreases with frequency. Such as in the ceramics with Ti^4+^ content of x = 0.4 ~ 0.6, the permittivity is apparently higher than that of the undoping one and decreases rapidly with increasing frequency at low frequency range, because the un-localized charges form in the barium ferrites with initially doping Ti^4+^ ions. Higher permittivity and more rapid decline at low frequency are exhibited with Ti^4+^ content increasing from x = 0.4 to x = 0.6. However, Fe^3+^ will most probably transform into Fe^2+^ to keep charge balance in the ferrites with high doping of Ti^4+^ ions. It implies that the electric charges generated will be localized between the two ions to form Fe^3+^ and Fe^2+^ pairs. Thus, as revealed in Fig. 6(a), the decline of permittivity at low frequency range becomes smoother with increasing Ti^4+^ content from x = 0.4 ~ 0.6 to x = 0.8.

In fact, the step-like shoulder at middle frequency range in BaFe_12-x_Ti_x_O_19_ with x ≥ 0.6 is based on Fe^3+^/Fe^2+^ pair dipoles. As analyzed above, Fe^2+^ ions are supposed to be abundantly produced in the ferrites since x reaches 0.6. Fe^3+^ and Fe^2+^ pairs make most probably electron pair dipoles in the ferrites. The higher the Ti^4+^ content is in the ceramics, the more the amount of pair dipoles is. So the step-like shoulder which is generated generally by relaxation dipoles such as Fe^3+^ and Fe^2+^ pairs appears initially in BaFe_12-x_Ti_x_O_19_ with x ≥ 0.6 and becomes more apparent in BaFe_11.2_Ti_0.8_O_19_ ceramic reasonably. Hence, the high permittivity is probably dominantly contributed by the Fe^3+^/Fe^2+^ pair dipoles in BaFe_12-x_Ti_x_O_19_ with x ≥ 0.6 instead of by charge response in the ferrites with x < 0.6. Moreover, considering polycrystalline ceramics in this case, conductivity inhomogeneity will appear in the ferrites due to the different electron hopping styles or hopping species in grains compared with those in grain boundaries. According to the Koop's opinions, the conductivity inhomogeneity contributes importantly the high permittivity^11^. The colossal permittivity which is about 100 k below 100 KHz and 20 k above 1 MHz appears hence in high Ti^4+^ doped ferrite ceramics of BaFe_11.2_Ti_0.8_O_19_ based on these two important contributions.

Furthermore, besides of high permittivity, the dielectric loss tangent of the ferrites decreases attractively with doping Ti^4+^ ions. The smallest dielectric loss of 0.2 is obtained in BaFe_11.2_Ti_0.8_O_19_ at ~10 kHz. It is much lower than that of percolative ferroelectric/ferromagnetic composite ceramics with both extraordinary dielectric and magnetic properties. In fact, in percolative ferroelectric/ferromagnetic composite ceramics, a great deal of space charges and other deficiencies will be produced in the interfaces between ferrite phases and ferroelectric phases due to the two different lattice structures. However, these deficiencies can be effectively eliminated in the single-phased ceramics as the grain boundaries are relatively perfectly matched among the identical lattice structures. Thus, the part of dielectric loss contributed by interfacial polarization is significantly decreased and low dielectric loss of only 0.2 appears in the single-phased barium ferrite ceramics. Apparently, single-phased ferrite ceramics doped with Ti^4+^ ions are potential multifunctional ceramics with both impressive magnetic and dielectric properties.

### Dual properties appearing universally in the single-phased ferrites with Fe^3+^/Fe^2+^ pairs

**Figure 8:**
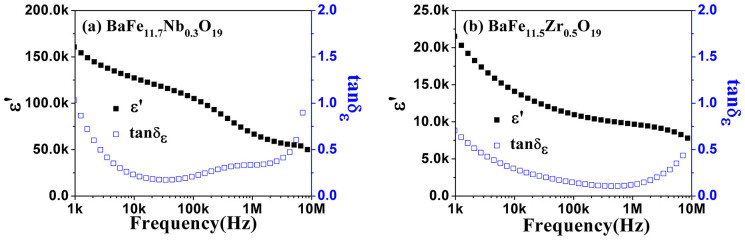
plots of the dielectric constant and dielectric loss of (a) BaFe_11.7_Nb_0.3_O_19_ and (b) BaFe_11.5_Zr_0.5_O_19_ as a function of frequency.

As a matter of fact, colossal permittivity as well as excellent magnetic properties appearing simultaneously in the single-phased ferrite ceramics is not due to Ti element itself but due to its higher valence state than that of Fe^3+^ in the ferrites. As is shown in Fig. 8, plots of the permittivity and dielectric loss tangent of Nb^5+^ and Zr^4+^ doped barium ferrite BaFe_11.7_Nb_0.3_O_19_ and BaFe_11.5_Zr_0.5_O_19_ as a function of frequency are exhibited respectively. Similarly, high value of permittivity which is over 50 k below 10 MHz and low dielectric loss tangent of about 0.18 around tens of kHz are obtained in BaFe_11.7_Nb_0.3_O_19_, and high value of permittivity of 10 k below 10 MHz and low dielectric loss tangent of about 0.11 around hundreds of kHz are obtained in BaFe_11.5_Zr_0.5_O_19_, respectively. A relaxation phenomenon is also clearly revealed for Nb^5+^ and Zr^4+^ doped barium ferrite ceramics. It indicates that different kinds of doping ions can be actually used to form the ferrites with dual susceptibilities and low losses, in which the only requirement is that the doping ions, such as Ti^4+^, Nb^5+^ and Zr^4+^, must have higher valence state than that of Fe^3+^ in the ferrites. It is worth noting that the ferrite and high valence ions used in this work are universal ones. That is to say, the single-phased ceramics with dual susceptibilities and low losses can be successfully and broadly obtained, which will benefit the area of developing electronic devices for integration and multi-functionality.

## Conclusions

In summary, single-phased ceramics of BaFe_12-x_Ti(Nb, Zr)_x_O_19_ with both considerable magnetic and dielectric properties were synthesized successfully by a sol-gel process. The Fe^3+^ and Fe^2+^ pair dipoles are produced by the substitution of high valence ions, such as Ti^4+^, Nb^5+^ and Zr^4+^, for Fe^3+^ based on charge balance in the ferrites. As Ti^4+^ substitutes preferably for Fe^3+^ in the sites with two compensated spin directions in the barium ferrite, the saturation magnetization and residual magnetization of the Ti^4+^ doped ferrites still keep high values to be practically used. Controlled by hysteresis loss, the magnetic loss of the Ti^4+^ doped ferrite ceramics diminishes effectively. Following the electron hopping between Fe^3+^ and Fe^2+^ ions and conductivity inhomogeneity between grains and grain boundaries in the ferrites, giant permittivity appears. Eliminating completely the interfaces between dissimilar phase structures, the dielectric loss tangent of the single-phased ferrites reduces significantly compared with that of the extensively interested percolative ferroelectric/ferromagnetic composite ceramics. Obviously, the single-phased ferrite ceramics doped by target ions in higher valence than that of Fe^3+^ reveal both extraordinary magnetic and dielectric properties simultaneously, which are even more competitive compared with the known systems such as multiferroic composites because of lower dielectric loss and thus become the most promising multifunctional materials in application of electronic devices for integration and multi-functionality.

## Methods

A series of BaFe_12-x_Ti_x_O_19_ ceramics with x varies from 0 to 0.8 were synthesized by a sol-gel process. Firstly, barium nitrate (Ba(NO_3_)_2_), ferric nitrate (Fe(NO_3_)_3_·9H_2_O), citric acid (C_6_H_8_O_7_·H_2_O) were weighted appropriately and dissolved in deionized water to obtain solutions A. Solutions B containing Ti^4+^ were obtained by dissolving Ti(OC_4_H_9_)_4_ and C_6_H_8_O_7_·H_2_O into anhydrous ethanol. According to stoichiometric proportion, the solutions A and B are mixed to get solutions C, ammonia was used to adjust the PH value to about 7. The solutions C were dried at 120°C in oven for 2 ~ 3 days to form fluffy dry gels, the gels were then further calcined at 800°C for 3 h and red-brown powders were achieved. Finally, the powders mixed with appropriate amount of 5% PVA were molded into a ring shape under a pressure of 10 Mpa and then sintered at 1200°C to form BaFe_12-x_Ti_x_O_19_ ceramics.

The phase structure and morphology of the ceramics were determined and observed by X-ray diffraction (XRD) (SHIMADZU XRD-6000, Cu Kα radiation) and scanning electron microscopy (SEM) (Hitachi SU-70 FESEM) respectively. The magnetic and dielectric properties were measured by magnetic property measurement system (MPMS-XL-5) and impedance analyzer (Agilent 4294A).

## Figures and Tables

**Table 1:** Structural parameters and volume of BaFe_12-x_Ti_x_O_19_ ceramics (x = 0, 0.4, 0.6 and 0.8) sintered at 1200°C for 3 h

x	a[Å]	c[Å]	V[Å^3^]
**0**	5.904	23.251	701.889
**0.4**	5.858	23.121	687.125
**0.6**	5.859	23.126	687.509
**0.8**	5.864	23.182	690.385

**Table 2:** Anisotropic field (*H*_a_), coercive force (*H*_c_), saturation magnetization (*M*_s_) and residual magnetization (*M*_r_) of the BaFe_12-x_Ti_x_O_19_ (x = 0, 0.4, 0.6 and 0.8) heated at 1200°C for 3 h

x	*H_a_* (KOe)	*H_c_* (KOe)	*M_s_* (emu/g )	*M_r_* (emu/g)
**0**	15.43	3.04	72.38	37.43
**0.4**	12.72	3.03	58.36	28.41
**0.6**	11.38	1.93	64.09	31.42
**0.8**	10.34	1.06	59.82	28.60
